# Performance of Active-Quenching SPAD Array Based on the Tri-State Gates of FPGA and Packaged with Bare Chip Stacking

**DOI:** 10.3390/s23094314

**Published:** 2023-04-27

**Authors:** Liangliang Liu, Wenxing Lv, Jian Liu, Xingan Zhang, Kun Liang, Ru Yang, Dejun Han

**Affiliations:** 1 College of Nuclear Science and Technology, Beijing Normal University, Beijing 100875, China; 2 Applied Optics Beijing Area Major Laboratory, Beijing Normal University, Beijing 100875, China

**Keywords:** single-photon avalanche diode (SPAD), active quenching circuit (AQC), field programmable gate array (FPGA), tri-state gate, 3D stacked package

## Abstract

The performance of an active-quenching single-photon avalanche diode (SPAD) array that is based on the tri-state gates of a field programmable gate array (FPGA) is presented. The array is implemented by stacking a bare 4 × 4 N-on-P SPAD array on a bare FPGA die, and the electrodes of the SPAD pixels and the I/O ports of the FPGA are connected through wire bonding within the same package. The active quenching action on each SPAD pixel is performed by using the properties of the tri-state gates of the FPGA. Digital signal processing, such as pulse counters, data encoders, and command interactions, is also performed by using the same FPGA. The breakdown voltage of the SPAD pixels, with an active area of 60 μm × 60 μm, is 47.2–48.0 V. When the device is reverse biased at a voltage of ~50.4 V, a response delay of ~50 ns, a dead time of 157 ns, a dark count rate of 2.44 kHz, and an afterpulsing probability of 6.9% are obtained. Its peak photon detection probability (PDP) reaches 17.0% at a peak wavelength of 760 nm and remains above 10% at 900 nm. This hybrid integrated SPAD array is reconfigurable and cost effective.

## 1. Introduction

The single-photon avalanche diode (SPAD) is a type of avalanche photodiode (APD) that operates in Geiger mode above the breakdown voltage, with a quenching circuit to stop the avalanche process. The electrical output of SPADs is binary, with a “0” or “1” output corresponding to whether photons are detected based on the fixed amplitude of the avalanche pulse. Due to their ultrahigh sensitivity—down to a single photon—and excellent photon counting and time-resolved performance, SPADs have gained significant attention in various fields [1]. In many applications, arrays of APDs are used, either in parallel to discriminate photon numbers (e.g., in silicon photomultipliers) [2] or with independent pixel outputs to enhance throughput [3].

When an APD detects a photon, it generates a macroscopic avalanche current through the process of impact ionization. This current can be self-sustaining and may block subsequent photon detection unless it is externally quenched. Therefore, a quenching circuit is critical for ensuring the optimal performance of the APD. It functions by rapidly extinguishing the avalanche current and resetting the bias voltage, allowing the APD to recover and detect subsequent photons.

In general, there are two types of quenching circuits: passive quenching circuits (PQCs) and active quenching circuits (AQCs) [4]. A PQC can be simply realized by serially connecting a ballast resistor with a high value (e.g., >50 kΩ) to the APD. However, the PQC does not have a well-defined quenching and recovery process but rather poor afterpulsing controllability. The AQC senses the rise of the avalanche pulse, synchronously generates a digital pulse to indicate the arrival of incident photons, and feeds back to a control circuit to lower the bias voltage of the APD below the breakdown voltage. At this time, the APD is turned off and is temporarily unable to detect photons. This hold-off state is usually maintained for a period of time to suppress the afterpulsing effect, and then the bias voltage is restored to its initial value (above the breakdown voltage). AQCs have been shown to offer superior performance and are commonly used in conjunction with single-photon avalanche diode (SPAD) arrays.

Most AQCs are implemented based on application-specific integrated circuits (ASICs) by either monolithic integration or hybrid integration, which has the advantages of strong device consistency and large-scale SPAD pixels integration [5,6,7]. However, these solutions have drawbacks, such as a long research and development cycle, a large initial investment, and difficulty upgrading. In other words, if the required quantity of the device is not large enough or the innovation of the device develops rapidly, it is obvious that ASIC-based AQCs are not cost effective. In contrast, the field programmable gate array (FPGA) is a logic device that can be reprogrammed and flexibly configured; it is complementary to ASICs, and a commercially available FPGA can replace an ASIC to achieve most of its functions to a certain extent [8]. Although SPAD arrays usually require FPGAs for external command interactions, image signal processors, power drivers, etc., and all these implementations largely rely on FPGA-based host boards, there are few reports on active quenching action directly realized by an FPGA. Samuel Burri et al. reported a compact linear SPAD camera system with commercial FPGA-based TDC modules for versatile 50 ps resolution time-resolved imaging; however, the variable-load quenching circuit used to implement the quenching process of the SPAD array was designed based on ASIC technology [9]. Subash Sachidananda et al. reported a reconfigurable hybrid design implemented by a System-on-Chip (SoC), which integrates an FPGA to vary the quench and reset parameters; however, it practically belongs to the ASIC-based AQC category [10].

This study presents a novel approach for implementing a SPAD array with an active quenching circuit based on the tri-state gates of a commercial FPGA. The proposed approach involves integrating a 4 × 4 N-on-P SPAD array with an FPGA using stacked die packaging, which is akin to the chiplet design concept [11]. The SPAD array serves to detect incident photons, while the FPGA carries out active quenching and data processing functions. This synergistic combination of the SPAD array with FPGA renders the pixels reconfigurable and facilitates real-time algorithmic implementations. Compared to discrete component-based AQCs for SPAD arrays, the proposed approach offers the advantages of compactness and performance consistency. Compared with ASIC-based AQCs for SPAD arrays, it is more cost-effective and offers greater flexibility. Therefore, this approach may be particularly well suited for low-investment scenarios, small production volumes, fast innovation, and pre-research stages.

The structure of this paper is organized as follows: In Section 2, we present the operational mechanism of the proposed SPAD array, which includes the logic principle of the active quenching circuit based on tri-state gates and the counting readout logic design of the SPAD array. In Section 3, we describe the implementation method of the SPAD array and its connection with the FPGA chip. The experimental results and their analysis are presented in Section 4. Finally, we summarize the conclusions and provide perspectives for future research in Section 5.

## 2. Operation Mechanism

**Figure 1:**
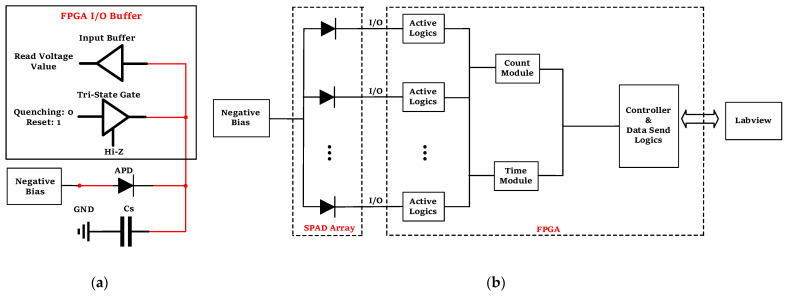
Schematic of the SPAD array based on the tri-state gate of the FPGA: (**a**) active quenching circuit for one of the SPAD pixels, where “Negative Bias” provides a direct current steady-state negative voltage and Cs is parasitic capacitance; (**b**) physical implementation of the SPAD array.

The tri-state gate is a fundamental component of an FPGA. Typically, the general-purpose input/output (GPIO) mechanism adopts the LVTTL level standard. The LVTTL level standard consists of a high-level voltage of 3.3 V and a low-level voltage of 0 V. In this study, the N electrode of an N-on-P SPAD is connected to a GPIO pin of the FPGA, and the P electrode (anode) is connected to a negative bias voltage slightly smaller than the breakdown voltage of the SPAD pixel. Initially, the output of the tri-state gate is set to high-level (3.3 V, “1”), and then it is switched to a high-impedance (“Hi-Z”) state. The read loop of the FPGA detects a high-level potential on the parasitic capacitor (Cs in Figure 1a), which is called the “complete” state. At this time, the potential of the N electrode of the SPAD remains high (3.3 V) because it is floating. The voltage between the N and P electrodes of the SPAD, i.e., −HV minus −3.3 V, is above the breakdown voltage, and the input buffer of the GPIO pin continuously reads the potential of the N electrode, which is called the “steady” state.

If a photon is incident on the SPAD, a photogenerated electron-hole pair (or a dark, thermally excited electron-hole pair) triggers an avalanche, and the SPAD is immediately discharged through the internal RC circuit. At the same time, the parasitic capacitance Cs charges the SPAD through the external loop. Then, the potential of the N electrode drops from the high level (3.3 V) to a lower value. Once the N electrode potential, read by the input buffer of the GPIO port, drops to a preset threshold voltage (e.g., 0.8 V in this study), the digital logic circuit inside the FPGA generates a logic pulse recording the arrival of incident photons and controls the tri-state gate to synchronously output a low potential level (0 V, “0”) to the N electrode. As a result, the bias voltage of the SPAD is quickly reduced below the breakdown voltage, and the avalanche is actively extinguished and maintained below the breakdown voltage for a certain amount of time (i.e., a hold-off time) to suppress the afterpulse, which is called the “hold-off” state. After the hold-off time, the digital logic circuit controls the tri-state gate to output a high-level voltage (3.3 V) for a certain amount of time to restore the potential of the N electrode to its initial value, which is called the “reset” state (due to the possibility of insufficient charging at the beginning of the reset, voltage fluctuations may occur at the parasitic capacitor). Then, the FPGA enters the “complete” state, and the readout circuit of the FPGA continuously detects the voltage value after power-up. It maintains the current state until the readout circuit detects a high voltage level, and the state transitions from “complete” to “steady”. This completes one logic cycle. The physical implementation of the SPAD array is schematically shown in Figure 1b. The readout logic circuits, such as the count module, time measure module, data encoder, and command interaction, are also provided by the logic resources of the FPGA.

**Figure 2:**
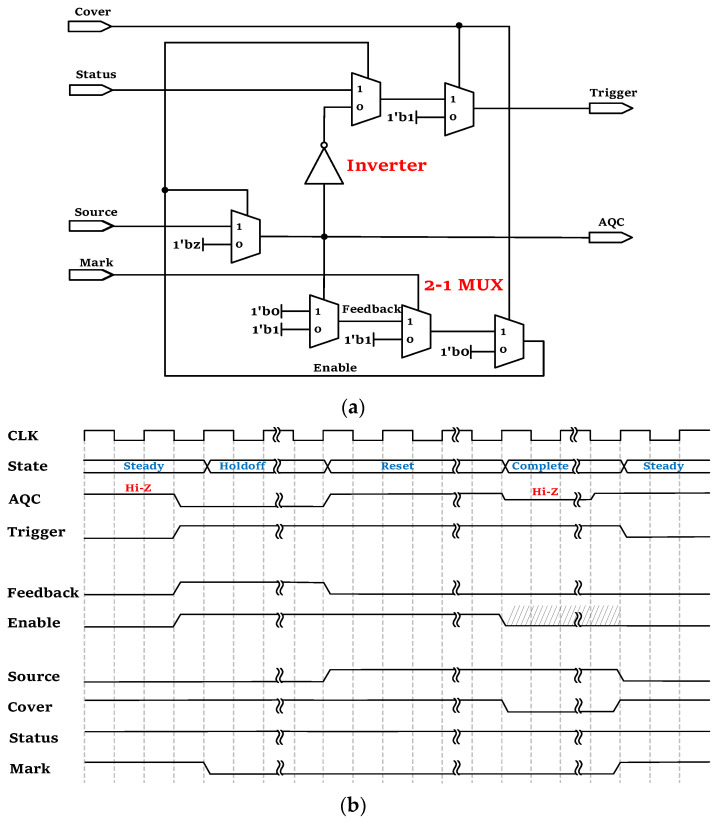
Diagram of active quenching logic in the FPGA: (**a**) logic function circuit of active quenching; and (**b**) sequential logic states of the whole quenching process.

The internal active quenching logic circuit design of the FPGA can be represented by a four-input and two-output block diagram, as shown in Figure 2. The four input parameters (Register Parameters), Cover, Status, Source, and Mark, work collaboratively to regulate the output states of the GPIO port, and their values are modified in accordance with the Finite State Machine (FSM) state transitions designed in FPGA. In detail, these four parameters control the internal logic to complete the functions required for different states, such as reset charging of the GPIO port, high voltage level stability detection, and hold-off time control.

The Trigger is a digital logic signal whose rising edge is synchronized with the moment when the avalanche current is actively quenched, and the AQC is the output value of the GPIO port. At the same time, two transition parameters are set, Enable and Feedback, which control the change in the tri-state gate. The Feedback control turns off the high-resistance state when the signal arrives and determines whether a transition to a low-potential-level state is needed to perform the quenching process, and Enable is the enabling parameter of the tri-state gate that controls the change between the high-impedance (“Hi-Z”) state and low-potential level to perform the quenching process. Figure 2a shows the combinational logic of the active quenching circuit when the photon arrives after the synthesis of FPGA, in which the transition of the tri-state gate from the “sensing” state to the “quenching” state is achieved by the combined logic of the inverter and the multiplexer. In Figure 2b, the working sequence diagram of the active quenching logic circuit is shown, in which CLK is a clock signal that indicates the timescale of state changes. In other words, it indicates the sequential relationship of the changes and settings of input and output parameters, as well as the transformation of the “complete”, “steady”, “hold-off” and “reset” states.

## 3. Implementation Method

**Figure 3:**
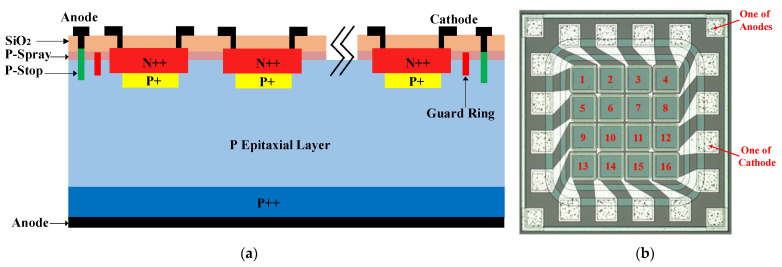
(**a**) Schematic structure. (**b**) Micrograph for the bare die of the 4 × 4 APD array.

The present work employed a 4 × 4 N-on-P type avalanche photodiode (APD) array designed by NDL (Novel Device Laboratory, Beijing, China) and fabricated by a foundry. Figure 3a shows the schematic structure of the APD pixels, where the P-Spray is P type to avoid surface inversion, the P-Stop is heavy P type to achieve ohmic contact for the front anode and prevent lateral spread of the depletion region for the PN junction of the guard ring to cutting edge, a guard ring is employed to prevent premature breakdown of the PN junction at the surface, the resistance and thickness of the epitaxial layer are 100 Ω∙cm and 33 μm, respectively, and the typical phosphorus and Born doping levels for the N++ and enrichment P+ are 5 × 10^15^ cm^−2^ and 3–4 × 10^12^ cm^−2^. Figure 3b is a micrograph of the bare die of the APD array, where the 16 APD pixels are labeled by sequence number. The active area of each APD pixel is 60 μm × 60 μm, and the size of the APD array is 0.65 mm × 0.65 mm. The 16 N-electrodes (i.e., cathodes) are led out to bonding pads separately, and the front P-electrodes (i.e., anodes) are led out to the bonding pads located at the four corners.

**Figure 4:**
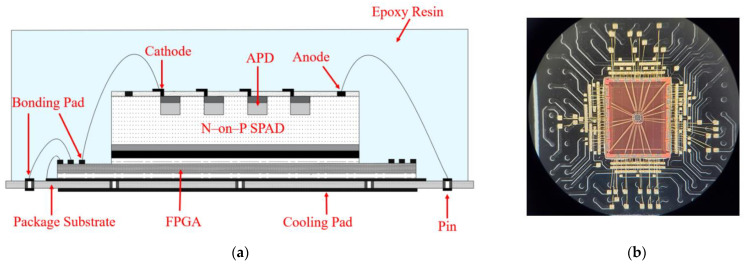
(**a**) Schematic structure. (**b**) Micrograph of the SPAD array sealed with transparent epoxy resin.

The bare SPAD array was integrated with a bare FPGA “GW2A-18” (Gowin Semiconductor Corporation, Guangdong, China) by a stacked die package [12]. As shown in Figure 4, the bare FPGA die with a size of 5.7 mm × 4.7 mm was first placed on the center of the package substrate, and the 4 × 4 bare SPAD array die was stacked on and bonded to the bare FPGA die with insulating glue. The lead-out pads of the N-electrodes were connected one-to-one to the lead-out pads of the GPIO pads on the FPGA by gold wire bonding. The lead-out pads of the P-electrodes on the front of the SPAD array were bonded to the package substrate directly, which provided the SPAD array with a negative bias voltage. All the other lead-out pads of the FPGA were wire bonded to the pads on the package substrate for power supply, ground connection and function configuration. Finally, the surface of the laminated dies was sealed by transparent epoxy resin (Epoxy Technology Inc, Billerica, MA, USA), and its transmittance at 400–1000 nm was greater than 99%. The area and thickness of the packaged SPAD array were 14 mm × 14 mm and 1.4 mm, respectively.

## 4. Results and Discussion

### 4.1. Characterization of the Static Properties

**Figure 5:**
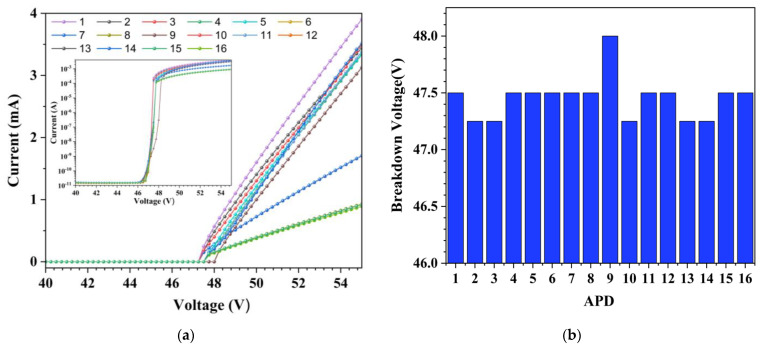
Representation of the (**a**) I–V curve in linear coordinates (the inset is in semilogarithmic coordinate form) and (**b**) breakdown voltage distribution for the 4 × 4 APD array.

The I-V curves of the APD pixels, obtained in a probe station with a Keithley 237 (Keysight Technologies Inc., Santa Rosa, CA, USA) Source Measure Unit (SMU) before packaging, are shown in Figure 5a. The first inflection point of the I-V curve is defined as the breakdown voltage (VB), and the reciprocal of the slope of the I-V curve above the breakdown voltage is regarded as the junction resistance, which is the sum of the depletion zone resistance and the neutral zone resistance and is distributed in the range of 2.1–8.4 kΩ. As shown in Figure 5b, the breakdown voltages of the 4 × 4 APD array are distributed in the range of 47.2–48.0 V. Figure 5 shows that the variations in both the breakdown voltages and the junction resistances have little relation with pixel positions, which can be attributed to the nonuniformity of the fabrication process and may have an impact on the overvoltage on each pixel. However, due to the digital feature of the APD pixels, the nonuniformity of the junction resistance had no significant influence on the performance of the device, such as digital signal generation and counting.

**Figure 6:**
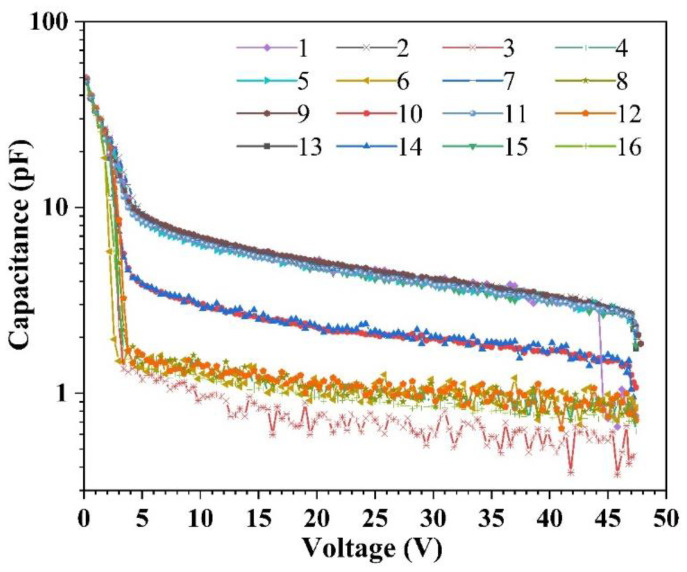
The C-V curves for the 16 APD pixels before packaging; the curves in the figure can be divided into three clusters. The top cluster corresponds to APD positions 1, 2, 5, 7, 9, 11, 13, and 15 in the APD array shown in Figure 3b. The middle cluster corresponds to APD positions 10 and 14, while the bottom cluster corresponds to APD positions 3, 4, 6, 8, 12, and 16.

In the graph presented in Figure 6, the logarithmic *y*-axis is employed to highlight the differences in capacitance among the 16 APD pixels prior to packaging. The capacitance-voltage (C-V) characteristics were measured using an Agilent E4980A precision LCR meter (Agilent Technologies Co. Ltd., Santa Clara, CA, USA), revealing junction capacitances that are distributed in the range of 0.45–2.24 pF near the breakdown voltage. Interestingly, there was no correlation between the capacitance values and the pixel position. However, the nonuniformity of both the junction capacitance and resistance may affect the consistency of the response time of APD pixels due to the variation in the resistance-capacitance (RC) time constant.

### 4.2. Characterization of Dynamic Properties

In this study, because the yield of packaging was not very high, not all SPAD cells could operate normally. One typical SPAD was chosen to verify the feasibility of the device concept presented in this manuscript. Its breakdown voltage was approximately 47.2 V, and the bias voltage on the P-electrode was set to −47.1 V. Therefore, the overvoltage was approximately 3.2 V when the N-electrode was provided with a high level of 3.3 V by the FPGA.

#### 4.2.1. Waveform

**Figure 7:**
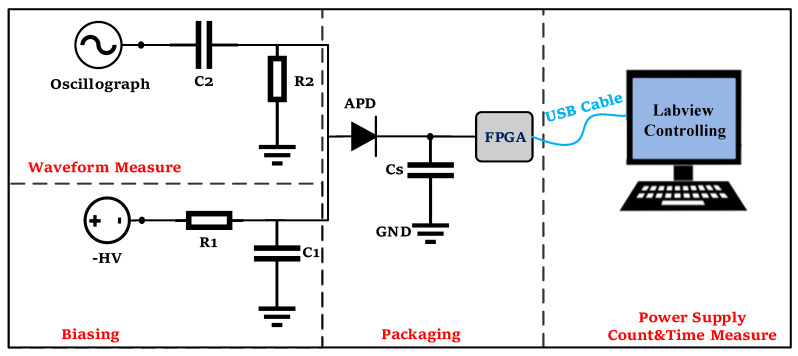
Experimental setup for measuring the pulse waveform, dark count, and afterpulse probability. The “biasing” loop is used to provide bias to the device, and R1 and C1 are used to create a low-pass filter circuit. The “waveform measure” loop is designed to monitor the active quenching process via waveform analysis from this output. R2 is the sampling resistance, and C2 is the capacitance that allows the AC signal to pass but not the DC signal. Cs represents the sum of the parasitic capacitances of the FPGA, SPAD, and their packaging. The “Count & Time Measure” loop is designed to measure count information combined with time information and send it to the personal computer. Typical values for R1, R2, C1, C2, and Cs are 500 Ω, 50 Ω, 1 µF, 10 nF, and ~4.4 pF, respectively.

The setup used to measure the pulse waveform is illustrated in Figure 7. The P electrode was connected to an adjustable voltage source Keithley 6430 (Keysight Technologies Inc., Santa Rosa, CA, USA) set to −47.1 V to provide the DC bias. To suppress power alternating current (AC) fluctuations, a low-pass filter circuit comprising R1 and C1 was employed. The AC signal, carried by capacitance C2, was sampled by resistance R2 and then outputted to an oscilloscope “LeCroy 640Zi” (Teledyne LeCroy Inc., Chestnut Ridge, NY, USA). Finally, the FPGA was connected to a personal computer to act as the power supply of the N-electrode.

**Figure 8:**
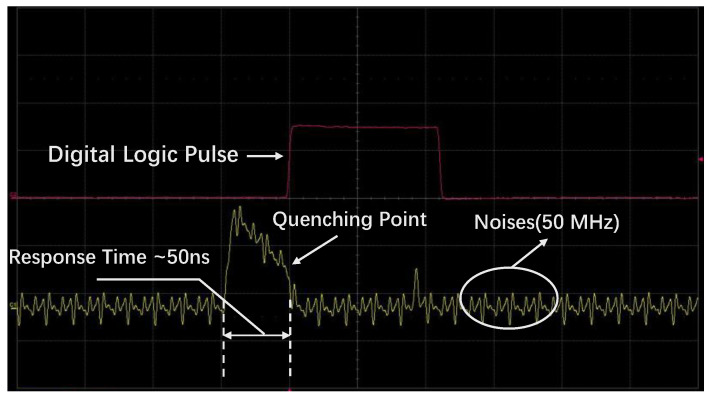
Output pulse of the P electrode (the yellow line) and generated digital logic pulse (the purple line) of an active quenching SPAD pixel.

Figure 8 displays the current pulse of the P electrode for the active quenching SPAD pixel. When an avalanche occurs on the SPAD pixel, the parasitic capacitance in the FPGA discharges rapidly through the P electrode, resulting in a rapid decrease in voltage across the two ends of the parasitic capacitance to a low level. Meanwhile, the voltage on the P electrode increases rapidly, as indicated by the first analog pulse of the P electrode labeled with a yellow line. The internal logic of the FPGA detects this potential change in parasitic capacitance, and when it falls below a preset threshold (0.8 V in this study), the FPGA generates a digital signal labeled with a purple line. The pulse width of ~50 ns for the analog pulse represents the time between photon impingement and a triggered response, which is referred to as the response time. The high-frequency noise of 50 MHz is caused by the crystal oscillator providing clock signals to the FPGA, but it has no influence on the SPAD operation, as shown by the digital logic pulse. Moreover, the avalanche information can be determined by extracting the digital logic pulse, and these counts can also be used to obtain a characterization of the device’s overall quality parameters, such as dark count, afterpulsing probability, and PDP. The response time primarily depends on the capacitance and resistance influence, and the input buffer of the FPGA detects the voltage at both ends of the parasitic capacitance (Cs in Figure 7) to determine the “1” and “0” status. The voltage at both ends of the parasitic capacitance decays exponentially when an avalanche occurs. The time constant of the exponential decay is the product of the total capacitance and the total resistance, where the total capacitance is the sum of the parasitic capacitance and the junction capacitance and the total resistance is the junction resistance. The device’s junction capacitance is approximately 0.45–2.24 pF, and the parasitic capacitance of the I/O buffer in the FPGA is approximately 4.4 pF, while the junction resistance is approximately 2.1–8.4 kΩ. Therefore, reducing the time constant will effectively decrease the response time in future studies.

#### 4.2.2. Dark Count and Afterpulse Probability

**Figure 9:**
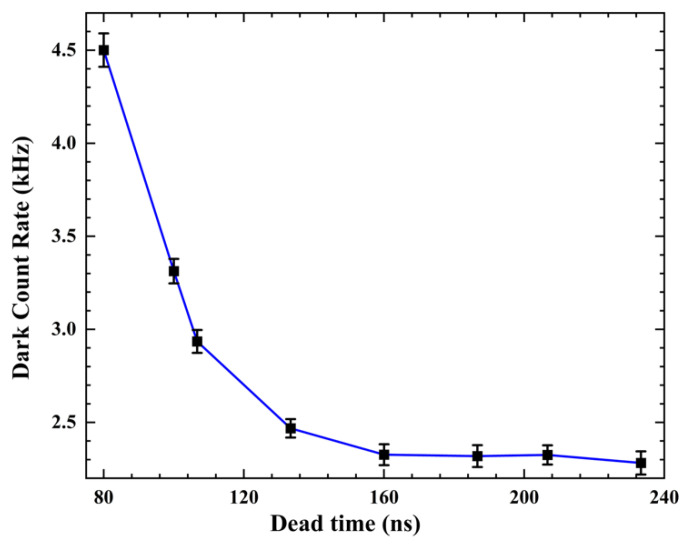
Dependence of the dark count rate on dead time.

The dark count rate refers to the number of avalanche pulses generated by a device due to various effects, such as thermal excitation, field-induced tunneling, optical crosstalk, and afterpulse, in the absence of any illumination. This parameter plays a crucial role in determining the signal-to-noise ratio. In our study, we measured the dark count rate using the counting module designed in the FPGA (shown as the Count Module in Figure 1b) without the need for an oscilloscope. The module sent the information to the LabVIEW control program on the computer via a USB cable. The device schematic is illustrated in Figure 7, where the “Waveform Measure” loop is not required during the measurement process. For the P electrode, we set the bias voltage to −47.1 V. In Figure 9, we show the relationship between the dead time (i.e., the sum of the hold-off time, response time, reset time and reset stability detection time) and the dark count rate of the SPAD pixel. We controlled the dead time of the device by adjusting the hold-off time, and the results show that the dark count rate decreased significantly as the hold-off time decreased, indicating effective suppression of the afterpulse. By adjusting the hold-off time to 84 ns, the dead time was approximately 157 ns, and the dark count rate was 2.44 kHz, with a response time of 50 ns, a reset time of 13 ns, and a reset stability detection time of 10 ns.

**Figure 10:**
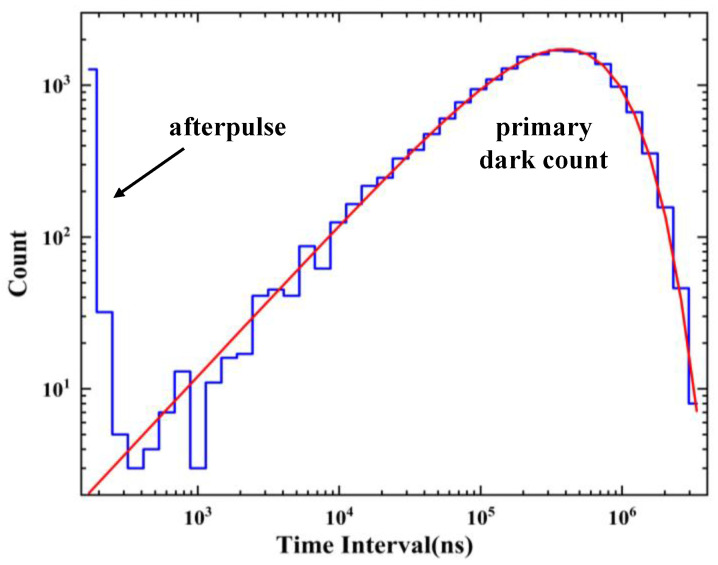
Statistical histogram of dark counts depending on time intervals in logarithmic coordinates at over voltage of 3.2 V and dead time of 157 ns; the location of the maximum corresponds to the inverse of the primary dark count rate, and the red line represents the Poissonian fit of the distribution of primary dark events.

Depending on whether the generation of dark counts occurs independently and randomly, dark count sources can be divided into two categories: primary dark counts and correlated noise. The correlated noise mainly comes from the afterpulse and optical crosstalk. The afterpulse occurs in the process of the delayed release of carriers trapped by defects in the bulk material after the avalanche pulse. The primary dark pulse mainly originates from thermal excitation and field-induced tunneling, and it satisfies the Poisson distribution. Therefore, the probability density of zero occurrences in time is in the form of exponential decay as presented in Equation (1) [13]. This paper represents the statistical histogram of the time interval in logarithmic form to more intuitively visualize the distribution of the afterpulse. The statistical calculation of the time interval is converted from the original [t, t+dt] to [log (*t*), log (*t*) + d[log(t)]]. As the probabilities that events will occur within equal time intervals dt are equal, the probability $y^{\prime }(t)$ of an event occurring at logarithmic coordinates can be expressed by Equation (3):

(1) $$y(t)=a\cdot e^{-b\cdot t}$$

(2) $$y(t)\cdot dt=a\cdot e^{-b\cdot t}\cdot \mathrm{dt}=y^{\prime }(t)\cdot d[\log(t)]=y^{\prime }(t)\cdot \frac{1}{t}\cdot dt$$

(3) $$y^{\prime }(t)=a\cdot t\cdot e^{-b\cdot t}$$

(4) $$P_{\mathrm{afterpluse}}=\frac{N_{\mathrm{afterpulse}}}{N_{\mathrm{primary}}}$$

where a is the normalization coefficient and b is the count rate of the primary SPAD dark pulse [14]. We fitted the statistical histogram in Figure 10 to visually show the distributions of the afterpulse and the primary dark pulse. The count outside the fitted curve (the part of the afterpulse in Figure 10) is recorded to represent the count of the afterpulse (indicated as $N_{\mathrm{afterpulse}}$), and the count of the fitted curve (the part of the primary dark count in Figure 10) is recorded to represent the count of the primary dark pulse (indicated as $N_{\mathrm{primary}}$). The afterpulsing probability is defined as the ratio of the afterpulsing count $N_{\mathrm{afterpluse}}$ to the primary dark pulse count $N_{\mathrm{primary}}$ in Equation (4).

**Figure 11:**
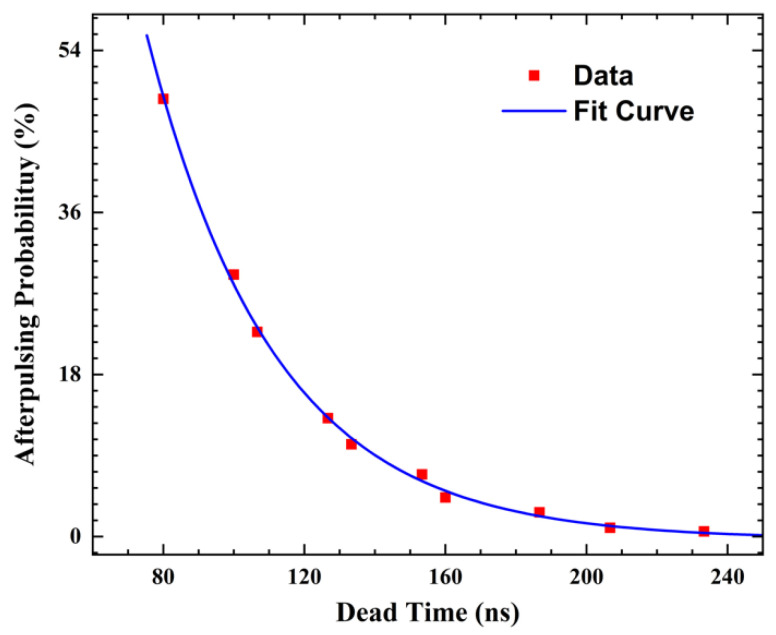
Variation in afterpulsing probability with dead time.

Due to the lack of a time digital conversion (TDC) module in the GW2A-18 bare FPGA chip, measuring the generation time of each pulse with ps-level accuracy is not possible using this FPGA. However, because the SPAD exhibits low dark count rates and the temporal precision requirement for measuring the afterpulse probability is relatively low, we employed a 300 MHz clock with a time accuracy of approximately 3.3 ns to obtain the time interval information of adjacent pulses. The time module designed in the FPGA (Figure 1b) was used to process the information, and the information was sent to the LabVIEW control program in the computer via USB cable for further processing, as depicted in Figure 7. The afterpulse probability density ${\;P}_{}\left(t\right)$ was expressed using Equation (5) with the double level model described in Refs [15,16]. The relationship between the dead time and afterpulse probability is analyzed in Figure 11, and the red curve represents the fitting curve of the afterpulse probability with the deadtime, expressed as Equation (6), which was used to explain the theoretical model of afterpulse generation in the fitting curve model.

(5) $$P_{}\left(t\right)=N_{\mathrm{apf}\;}/\tau_{\mathrm{apf}\;}\cdot e^{-t/\tau_{\mathrm{apf}\;}}+N_{\mathrm{aps}\;}/\tau_{\mathrm{aps}\;}\cdot e^{-t/\tau_{\mathrm{aps}\;}}$$

(6) $$P_{}\left(t_{N}\right)=\int_{t_{N}}^{\infty }\left(N_{\mathrm{apf}\;}/\tau_{\mathrm{apf}\;}\cdot e^{-t/\tau_{\mathrm{apf}\;}}+N_{\mathrm{aps}\;}/\tau_{\mathrm{aps}\;}\cdot e^{-t/\tau_{\mathrm{aps}\;}}\right)dt=N_{\mathrm{apf}\;}\cdot e^{-t_{N}/\tau_{\mathrm{apf}\;}}+N_{\mathrm{aps}\;}\cdot e^{-t_{N}/\tau_{\mathrm{aps}}}$$

Here, the fit quality can be significantly improved by using two different time constants, $\tau_{\mathrm{apf}\;}$ and $\tau_{\mathrm{aps}}$, one describing a fast component of afterpulse generation and the other a slow one. $N_{\mathrm{apf}\;}$ and $N_{\mathrm{aps}\;}$ correspond to the integrated numbers of fast and slow afterpulses, respectively, $t_{N}$ represents the value of the dead time. However, due to the long response time, the time required for reset, and the high voltage level stability detected (“complete” state) after resetting, the fast component of afterpulse generation could not be obtained. The release time of the slow component of afterpulse generation was determined to be approximately 36.1 ns through fitting, which is determined by the material and structure of the detector. Furthermore, it was observed from Figure 11 that the hold-off time drops below 7% with a dead time greater than 157 ns.

#### 4.2.3. Photon Detection Probability and Linear Response Dynamic Range 

**Figure 12:**
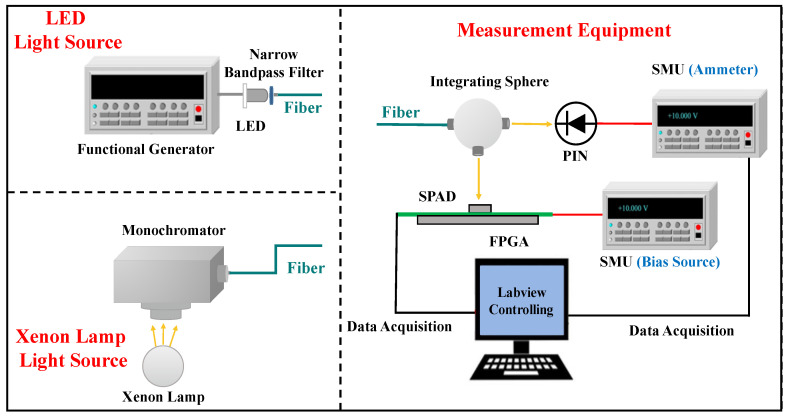
Experimental setup for the measurements of the photon detection probability and dynamic range. The dynamic range measurement uses an LED light source, and the PDP measurement uses a xenon lamp light source.

In this study, the photon counting method was utilized to measure the photon detection probability (PDP) of the SPAD pixel [17]. The experimental setup included measurement equipment and a light source, as shown in Figure 12, and this method used a xenon lamp light source “LSH-X 150 W” (ZOLIX INSTRUMENTS Co. Ltd, Beijing, China). This light source with a monochromator was employed to provide a tunable monochromatic light source. Regarding the measurement equipment, the current value of a calibrated PIN photodiode “Hamamatsu S1227-33BQ” (Hamamatsu Photonics Co., Ltd., Shizuoka, Japan) measured by SMU “Keithley 2635B” was used to monitor the intensity of light impinging on the SPAD pixel. Another SMU (Keithley 6430) was used to provide a negative bias to the SPAD. The count information of the SPAD was observed with the counting module in FPGA and sent to the computer for further processing.

**Figure 13:**
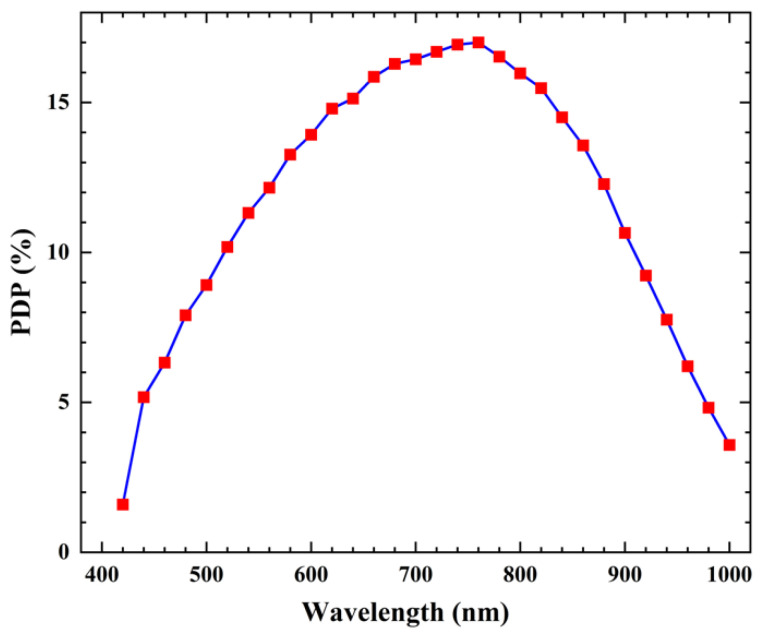
Dependence of the photon detection probability on the wavelength.

The photon detection probability (PDP) is defined as the product of the quantum efficiency and the avalanche triggering probability. By setting an adequate long dead time (~157 ns), the afterpulse probability was approximately 6.9%. Under these conditions, a relatively accurate measurement of the photon detection probability (PDP) could be obtained for the pixel. Figure 13 depicts the wavelength dependence of the PDP. At a bias voltage of −47.1 V on the P-electrode, the PDP reached 17.0% at a peak wavelength of 760 nm and remained above 10% at 900 nm.

For the measurement of dynamic range, a similar setup to that used for PDP measurement was employed. However, a light emitting diode (LED) was used instead of the xenon lamp light source, as the SPAD dynamic response range was still not saturated at the maximum intensity of the xenon lamp. The LED light source had a wavelength of 770 nm, and a schematic diagram of the LED light source and measurement equipment is shown in Figure 12.

**Figure 14:**
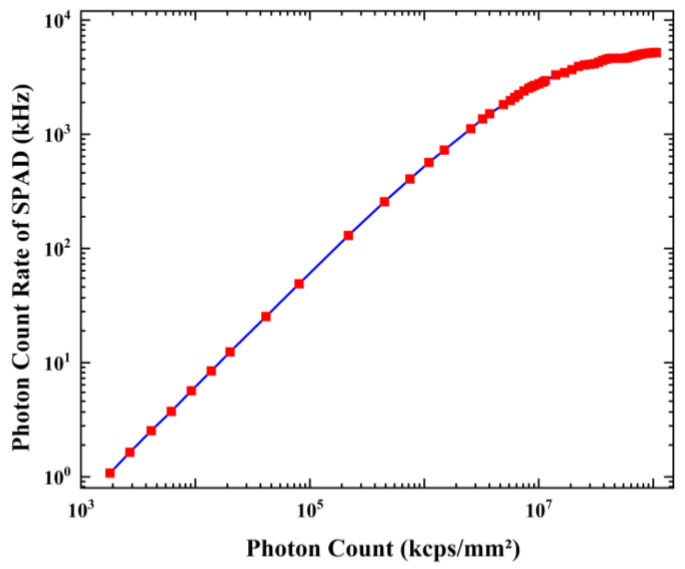
Dependence of the net photon count rate of the SPAD on the photon count per unit time per square millimeter.

Figure 14 illustrates the relationship between the net photon count rate (i.e., the photon count rate minus the dark count rate) and the photon count per unit time per square millimeter incident on the SPAD (indicated as $n_{\mathrm{ts}}$) at a bias voltage of −47.1V on the P-electrode (corresponding to a bias voltage across the SPAD of 50.4 V). Here, $n_{\mathrm{ts}}$ is obtained by Equations (7)–(9):

(7) $$R\cdot \frac{\mathrm{nh}\nu }{t}=I$$

(8) $$\frac{n}{t}=\frac{I}{R\cdot h\cdot \nu }=\frac{I\cdot \lambda }{R\cdot h\cdot c}$$

(9) $$n_{\mathrm{ts}}=\frac{n}{t\cdot s}\cdot \frac{\mathrm{lf}\left(\mathrm{SPAD}\right)}{\mathrm{lf}\left(\mathrm{PIN}\right)}=\frac{I\cdot \lambda }{R\cdot h\cdot c\cdot s}\cdot \frac{\mathrm{lf}\left(\mathrm{SPAD}\right)}{\mathrm{lf}\left(\mathrm{PIN}\right)}$$

where n represents the number of photons from the output port (PIN port) of the integrating sphere; I represents the photocurrent; R represents the photon responsivity of the abovementioned calibrated PIN at a 770 nm wavelength, which is approximately 0.37082; λ represents the LED emission wavelength of approximately 770 nm (FWHM of 10 nm); h represents the Planck constant; ν represents the frequency of light; s represents the area of the PIN, which is 5.7 mm^2^; and $\frac{\mathrm{lf}(\mathrm{SPAD})}{\mathrm{lf}(\mathrm{PIN})}$ represents the ratio of light flux at both ports, which is 98:100. Hence, the discrepancy between the horizontal and vertical coordinates in Figure 14 is primarily dependent on the effective area of the SPAD (0.0036 mm^2^) and the PDP (~16.5%). The light flux ratio was calibrated by utilizing the same PIN at both ends of the integrating sphere, which is explained in Ref [16].

The graph shows an approximately three-order linear dynamic range, ranging from 2.44 kHz to 2.11 MHz. The lower limit of the dynamic range is determined by the dark count rate, while the upper limit is limited by the dead time (~157 ns) of the SPAD pixel. Although the upper limit of the dynamic range can be increased by reducing the hold-off time, this would worsen the lower limit by increasing the dark count rate. In future studies, the dynamic range could be effectively improved by decreasing the response time.

## 5. Conclusions and Perspectives

A preliminary active quenching SPAD array based on the tri-state gates of an FPGA was demonstrated. It was implemented by stacking a bare 4 × 4 N-on-P SPAD array on a bare FPGA die, and the electrodes of the SPAD pixels and the I/O ports of the FPGA die were connected through wire bonding within the same package. Both the active quenching action and digital signal processing were realized by the same FPGA. The integration of the FPGA with a SPAD array allows for logical reconfiguration based on the specific applications; i.e., it not only allows the reconfiguration of SPAD array operation and quenching control but also allows the reconfiguration of subsequent digital signal processing by using the same hardware. Such flexibility enables real-time algorithmic implementation. Compared to the discrete component-based AQC SPAD array, it has the merits of compactness and performance consistency. Compared to the ASIC-based AQC SPAD array, it is cost effective and flexible as well as suitable for low investment, small production volumes, fast innovation, and pre-research stages.

Stacking a bare SPAD array on a bare FPGA is beneficial because it uses the GPIO pins of the FPGA as much as possible and makes the device as compact as possible. However, bare FPGA dies are very difficult to obtain. As we were limited by the timing function of the bare die employed in this study, the response time resolution of the device was not investigated. In the next step, we will implement the active quenching SPAD array by wire bonding a bare SPAD array with a commercial packaged FPGA via a printed circuit board (PCB), and the trade-off between timing performance and achievable pixel number will be considered.

## Data Availability

No new data were created or analyzed in this study. Data sharing is not applicable to this article.
